# The Characteristics of Microbiome and Cytokines in Healthy Implants and Peri-Implantitis of the Same Individuals

**DOI:** 10.3390/jcm11195817

**Published:** 2022-09-30

**Authors:** Lu Song, Jimin Jiang, Jia Li, Chuan Zhou, Yanqi Chen, Hongye Lu, Fuming He

**Affiliations:** Stomatology Hospital, School of Stomatology, Zhejiang University School of Medicine, Zhejiang Provincial Clinical Research Center for Oral Diseases, Key Laboratory of Oral Biomedical Research of Zhejiang Province, Cancer Center of Zhejiang University, No. 166, QiuTao Rd (N), Shangcheng District, Hangzhou 310020, China

**Keywords:** peri-implantitis, microbiota, cytokines, biomarkers

## Abstract

Aim: To characterize the profile of submucosal microbiome and cytokine levels in peri-implant crevicular fluid (PICF) from clinically healthy implants and peri-implantitis in the same individuals. Material and Methods: A total of 170 patients were screened and, finally, 14 patients with at least one healthy implant and one peri-implantitis implant were included. Submucosal microbiota and cytokines from 28 implants were analyzed using 16S rRNA gene sequencing and multifactor assays, respectively. Correlations of clinical indexes and microbiota or cytokines were analyzed using Spearman’s correlation coefficient. A random forest classification model was constructed. Results: Peri-implantitis sites harbored higher microbial diversity, as well as more Gram-negative bacteria and anaerobic bacteria, compared with healthy implants sites. The genera of *Peptostreptococcaceae XIG-1*, *Treponema*, *Porphyromonas*, and *Lachnospiraceae G-8*, as well as the cytokines of IL-17A, IL-6, IL-15, G-CSF, RANTES, and IL-1β were significantly higher in peri-implantitis than healthy implants. Furthermore, these genera and cytokines had positive relationships with clinical parameters, including probing depth (PD), bleeding on probing (BOP), and marginal bone loss (MBL). The classification model picked out the top 15 biomarkers, such as IL-17A, IL-6, IL-15, VEGF, IL-1β, *Peptostreptococcaceae XIG-1*, *Haemophilus*, and *Treponema*, and obtained an area under the curve (AUC) of 0.85. Conclusions: There are more pathogenic bacteria and inflammatory cytokines in peri-implantitis sites, and biomarkers could facilitate the diagnosis of peri-implantitis.

## 1. Introduction

Peri-implantitis is a complicated polymicrobial biofilm-induced inflammatory process associated with supporting bone loss, and it is commonly reported as one of the major contributors to implant failure [1]. Epidemiological research showed that the prevalence rate of peri-implantitis exceeds 20% [2]. Untreated peri-implantitis progresses faster in a non-linear accelerating pattern due to the collagen fibers in a parallel orientation and poor vascularity in peri-implant connective tissue [3,4]. Moreover, the prognosis of peri-implantitis therapy is unsatisfactory, and a quarter of implants with peri-implantitis are lost within five years, even after systemic treatment [5]. Thus far, the pathogenesis, diagnosis, and therapeutic strategies for peri-implantitis have received widespread attention, but have not yet been resolved.

Recent studies have elucidated that the primary etiologic factor of peri-implantitis is microbial biofilm accumulation, and microorganisms with related virulence factors could trigger infection and inflammatory response of the host, causing a series of immunological reactions [6]. Gaining more insights into the peri-implantitis-associated microbiome might facilitate investigating the etiology of diseases. Previous studies that analyzed the microbial profiles of peri-implantitis relied on anaerobic culture-based techniques and close-ended molecular approaches, which could only focus on specific bacteria and preclude the identification of potentially relevant microbiota that were not targeted by the technique [7]. Now, 16S rRNA gene amplicons sequencing has emerged and could expand the catalog of bacterial taxa with high resolution [8]. Kumar et al. [9] were the first to sequence 16S rRNA gene amplicons from healthy implant sites and peri-implantitis sites, and their results identified predominant abundant microbiota at all taxonomic levels. In subsequent studies, some researchers also identified specific microbial signatures between healthy and diseased implant sites [10,11,12]; however, there is no clear consensus on the microbiota composition of peri-implantitis among these studies, and thorough profiling of the microbiome with differential diagnoses in the same patients is needed [13].

Simultaneously, the transformation of healthy peri-implant sulcus into the peri-implantitis pocket is induced by the exceeding host inflammatory response that is triggered by dysbacteriosis, and immune-derived mediators may facilitate alveolar bone resorption either directly or indirectly [14]. Cytokine levels of peri-implant crevicular fluid (PICF), which could be obtained conveniently and non-invasively, may reflect the situations of healthy or diseased peri-implant sites [15]. Recent studies have focused on some common pro-inflammatory cytokine levels, such as IL-1β, TNF-α, and IL-6, which could exert synergistic properties in the initiation of inflammatory marker cascade and bone resorption, and showed these cytokines were higher in the peri-implantitis sites compared to healthy implant sites [16]. However, several important chemokines and growth factors have scarcely been performed properly in previous research [17], and whether these cytokine levels assist in the diagnosis and therapeutic strategies of peri-implantitis needs to be further investigated. Moreover, cytokines are involved in broad networks, which to a great extent orchestrate the immuno-inflammatory process [18]. A comprehensive profile of cytokines to explore the pathogenesis of peri-implantitis more profoundly is necessary.

Therefore, this clinical pair-matching cross-sectional study aimed to characterize the profile of submucosal microbiome and cytokine levels in PICF from healthy implants and peri-implantitis with 16S rRNA gene sequencing and multifactor assays, which could monitor individual differences and confounders. A global profile of the peri-implant microenvironment could help better explore the pathology and therapeutic strategies for peri-implantitis.

## 2. Materials and Methods

### 2.1. Patients Recruitment and Data Collection

The study design was approved by the Ethics Committee of the Affiliated Stomatology Hospital, Zhejiang University School of Medicine, Zhejiang, China (Prot. Number 2021-66), and conducted according to the Declaration of Helsinki revised in 2008. The cross-sectional study followed the guidelines of Strengthening the Reporting of Observational Studies in Epidemiology (STROBE guideline) accessible through the EQUATOR network.

Patients were recruited in the Affiliated Stomatology Hospital, Zhejiang University School of Medicine. All patients were fully informed of this study and signed informed consent. Patient information, including age, gender, smoking habit, history of treatment, implant position, implant system, bone augmentation, and prosthetic types, was collected. Clinical indexes, including the plaque index (PLI), gingival index (GI), pocket depth (PD), presence of bleeding on probing (BOP), keratinized gingiva width (KGW), and marginal bone loss (MBL), were recorded. BOP was defined as the presence or absence of gingival bleeding within 15 s after probing with the implant-supported prostheses in place or removing the implant-supported prostheses based on the implant status, and MBL was measured by panoramic radiographs and cone-beam-computed tomography (CBCT) scanning. The diagnostic criteria were entirely based on the consensus of the 2017 World Workshop [19]. Peri-implant health was diagnosed with an absence of soft tissue inflammation and further additional bone loss following initial healing according to radiographic examination at baseline and at follow-up. Peri-implantitis was diagnosed with bone loss and increasing PD following initial healing, or with MBL ≥ 3 mm and PD ≥ 6 mm without previous examination data. Patients with peri-implantitis implants were treated after sample collection by an experienced dentist according to the CIST guideline [20]. The inclusion criteria were as follows: (1) ≥18 years of age; (2) Functional loading of implants over a year; (3) With at least one healthy implant and one implant diagnosed as peri-implantitis; (4) Understanding and giving informed consent. The exclusion criteria were as follows: (1) Pregnant or lactating women; (2) Suffering from uncontrolled systemic diseases (cardiovascular disease, kidney disease, diabetes, autoimmune deficiency syndrome, hepatitis, etc.); (3) Patients who had taken antibiotics, bisphosphonates, steroids, or non-steroidal anti-inflammatory drugs in the last three months; (4) Patients who had received periodontal treatment or peri-implant treatment in the last three months; (5) Severe smoking habit (smoking more than 20 cigarettes per day); (6) Suffering from mental illness, non-compliant patient.

### 2.2. Sample Collection and Processing

Samples were collected according to previous literature [21,22]. Briefly, before sample collection, patients were asked to rinse their mouths to remove food residue in the mouth with water. The peri-implant supragingival plaque was carefully removed with a scaler or small cotton ball, and the sample site was isolated with cotton rolls and dried with a gentle stream of air. Sterile paper was gently inserted apically into six sites, including mesial, middle, and distal sites on both the buccal and lingual sides, until a slight resistance was felt (≤25 N), and held for the 30 s. Samples contaminated with blood or saliva were discarded. The samples were immediately placed in a labeled sterile tube and processed with the casing (pipe-in-pipe) method [22]. After that, the samples were divided into supernatants and precipitation, and stored at −80 °C before subsequent analysis.

### 2.3. Cytokine Assessment in the PICF

The supernatant samples were processed using a multifactor analysis according to the manufacturer’s recommendations (Luminex^®^ Human 44 Cytokine Fixed Panel, #LKTM014, R&D System, Minneapolis, MN, USA). The results were analyzed using the Luminex 200 fluorescent detection system. The cytokines in PICF were estimated from the standard curve using the Millipore software.

### 2.4. DNA Extraction and Sequencing

Bacterial genomic DNA was extracted from the precipitation using a TIANamp Micro DNA Isolation Kit (TIANGEN BIOTECH, Beijing, China) and tested by a NanoDrop2000 spectrophotometer (Thermo Fisher Scientific, Inc., Waltham, MA, USA). The primers 338F (5′-ACTCCTACGGGAGGCAGCAG-3′) and 806R (5′-GGACTACNNGGGTATCTAAT-3′) were used to amplify the V3-V4 hypervariable region of the bacterial 16S rRNA gene. PCR products were purified using an Agencourt AMPure XP Kit (Beckman Coulter, Inc., Pasadena, CA, USA). Deep sequencing was performed on the Miseq PE300 platform (Allwegene Company, Beijing, China). After running, image analysis, base calling, and error estimation were performed using Illumina Analysis Pipeline (V2.6, San Diego, CA, USA).

### 2.5. Data Processing and Statistical Analysis

The raw data were screened, and sequences were removed from consideration if they were shorter than 200 bp or had a low-quality score (≤20). Qualified reads were clustered into operational taxonomic units (OTUs) at a similarity level of 97% using the Uparse algorithm of Vsearch (V2.7.1) software (Edgar, 2013). The BLAST tool (V2.6.0) was used to classify all sequences into different taxonomic groups against the Human Oral Microbiome Database (HOMD) (https://www.homd.org, accessed on 9 February 2021). The differences of microbial richness (Chao1) and diversity (Simpson) were analyzed by a Wilcoxon signed-rank paired test. Principal coordinates analysis (PCoA) based on weighted UniFrac distance was used to examine the similarity between different samples. Analysis of similarities (ANOSIM) was performed to compare the similarities between the two sites. Different taxon at all levels between healthy implants and peri-implantitis were analyzed with a Wilcoxon signed-rank paired test (*p* < 0.05) using the STAMP software [23]. Linear discriminant analysis effect size (LEfSe) analysis was used to present differential taxa between healthy implants and peri-implantitis, with a linear discriminant analysis (LDA) score threshold of 4.0. The phenotype structures of the microbiome were predicted based on 16S data using BugBase (https://bugbase.cs.umn.edu/index.html, accessed on 9 February 2021). SPSS (V26.0) was used for statistical analysis. The cytokine levels in healthy implant sites and peri-implantitis sites were analyzed with a Wilcoxon signed-rank paired test. A *p* < 0.05 and a false discovery rate (FDR, *q* < 0.05) were considered to be significantly different. The logarithm of fold change (FC) was computed. Correlations between the microbiome and cytokines and clinical indexes, including mean PD, BOP, and MBL, were analyzed using Spearman’s correlation coefficient. A random forest classification model was constructed, which was verified by the receiver operating characteristic (ROC).

## 3. Results

### 3.1. Demographic and Clinical Characteristics of the Study Population

The study was designed as a pair-matching cross-sectional study to compare healthy implants and peri-implantitis within the same individual. A total of 170 patients who had at least two implants were screened, with only 22 patients contributing 44 samples from healthy and peri-implantitis implants for eligibility criteria. However, some samples were excluded due to low concentration. Eventually, 28 samples from 14 patients were analyzed, and these samples achieved a power of 0.8 and α of 0.05, with an effect size of 0.7, using G * power software (V3.1).

The demographic characteristics of the clinical features of implants are shown in Table 1. The mean PD of peri-implantitis implant sites was significantly deeper than that of healthy implant sites (*p* < 0.01). The total volumes of PICF in the peri-implantitis sites were higher than in the healthy implant sites (*p* < 0.01). The detailed characteristics of every implant were listed in Tables S1 and S2, respectively.

### 3.2. Microbial Profile of Healthy Implant Sites and Peri-Implantitis Sites

**Figure 1:**
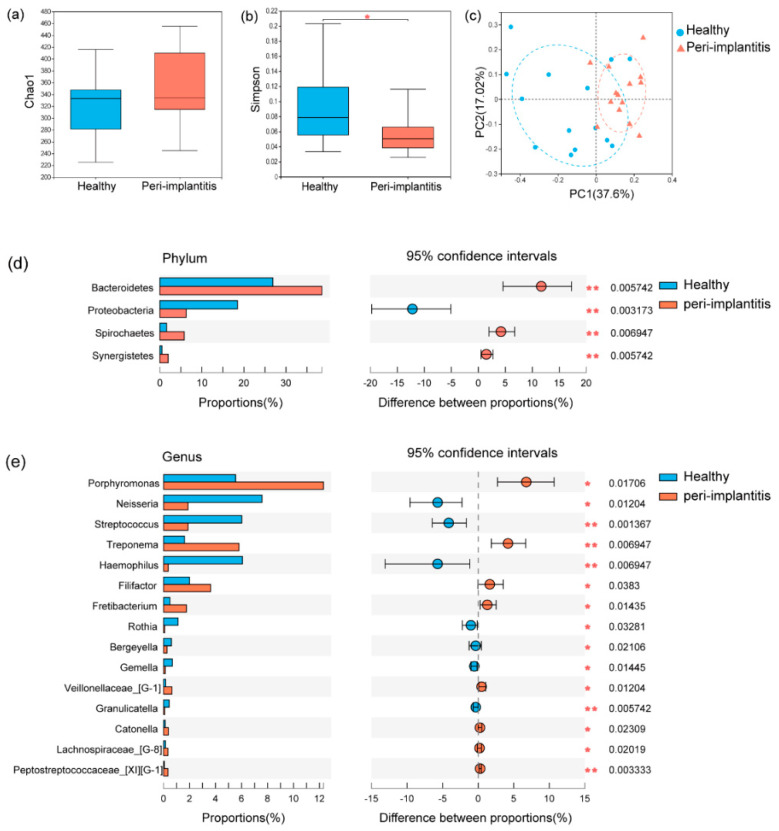
Microbial profile of healthy implants and peri−implantitis. The microbial richness presented with Chao1 (**a**) and microbial diversity presented with Simpson (**b**) were analyzed with a Wilcoxon signed−rank paired test. (**c**) Principal coordinate analysis (PCoA) based on Unifrac distance measurements showed the difference between healthy implants and peri−implantitis (*p* = 0.002, R = 0.26). The microbial composition of healthy implants and peri−implantitis was explored in terms of the relative abundances at the phylum level (**d**) and genus level (**e**) using the Wilcoxon signed−rank paired test. * *p* < 0.05, ** *p* < 0.01.

In this study, the sample sizes were sufficient for the microbiological analysis according to the species accumulation curve (Figure S1). A total of 751 OTUs from 28 samples were required, being clustered into 12 phyla, 29 classes, 48 orders, 77 families, 146 genera, and 353 species. The rarefaction curves (Figure S2) showed sufficient sequencing depth was reached in this study. The comparison of alpha diversity was analyzed between healthy and peri-implantitis sites. Microbial richness presented by Chao1 (Figure 1a) was not significantly different between healthy implants and peri-implantitis (*p* = 0.21), while microbial diversity presented by Simpson (Figure 1b) was significantly higher in peri-implantitis compared with healthy implants (*p* = 0.047). PCoA analysis (Figure 1c) based on Unifrac distance measurements showed a significant difference between healthy implants and peri-implantitis (*p* < 0.05), which indicated peri-implantitis harbored distinct microbiota from healthy implants. ANOSIM analysis showed that intergroup difference was significantly higher than intragroup difference (*p* = 0.002, *R* = 0.26), which confirmed the differential microbiota between healthy implants and peri-implantitis.

**Figure 2:**
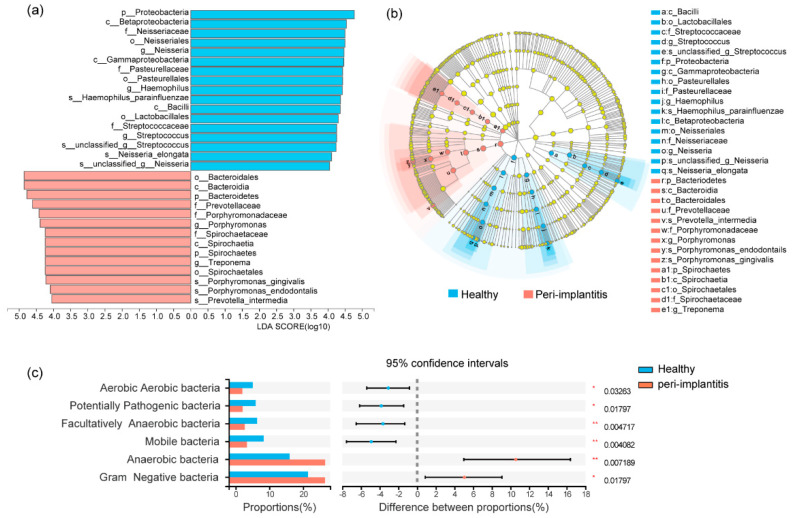
Linear discriminant analysis effect size (LEfSe) analysis of the healthy implants and peri−implantitis. (**a**) Linear discriminant analysis (LDA) represented statistical and biological differences between healthy implants and peri−implantitis (LDA > 4.0, *p* < 0.05). (**b**) The cladogram showed microbial differences between healthy implants and peri−implantitis at all phylogenic levels. (**c**) The predicted phenotype of the microbiota between healthy implants and peri−implantitis. * *p* < 0.05, ** *p* < 0.01.

Distinct microbiota between healthy implants and peri-implantitis were analyzed with pair-wise methods (Wilcoxon signed-rank test, *p* < 0.05). As shown in the bar plot, the phyla of *Bacteroidetes*, *Spirochaetes*, and *Synergistetes*, as well as the genera of *Porphyromonas*, *Treponema*, *Filifactor*, *Fretibacterium*, *Lachnospiraceae G-8*, and *Peptostreptococcaceae XIG-1*, were more abundant in peri-implantitis sites, while the phyla of *Proteobacteria*, as well as the genera of *Neisseria*, *Streptococcus*, *Haemophilus*, and *Rothia*, accounted for the main part of the submucosal microbiome in healthy implant sites (Figure 1d,e). Differential taxa at the class, order, family, and species levels between healthy implants and peri-implantitis are shown in Figures S3 and S4. LEfSe analysis was used to present the differential taxa ranging from phylum to species levels between healthy implants and peri-implantitis. As shown in the bar plot (Figure 2a) and cladogram (Figure 2b), *Porphyromonas gingivalis*, *Porphyromonas endodontails*, and *Prevotella intermedia* were more prevalent in peri-implantitis than healthy implants.

The phenotype structures of the microbiota were predicted. Gram-negative bacteria and anaerobic bacteria were significantly enriched in peri-implantitis (*p* < 0.05), while Gram-positive bacteria, aerobic bacteria, facultatively anaerobic bacteria, mobile bacteria, and potentially pathogenic bacteria were abundant in the healthy implants (*p* < 0.05) (Figure 2c).

### 3.3. Cytokine Levels in PICF of Healthy and Peri-Implantitis Sites

The cytokines that had significant differences (*p* < 0.05) between healthy implant and peri-implantitis sites are shown in Table 2, and the cytokines with no difference are shown in Table S3. Some pro-inflammatory cytokines were significantly higher in peri-implantitis compared to healthy implants, including IL-1β (1403.0 (735.8, 2083.8) pg/mL vs. 4311.0 (2388.5, 5502.5) pg/mL; *p* < 0.001; *q* = 0.004), IL-6 (5.4 (4.5, 8.2) pg/mL, vs. 38.3 (16.2, 221.7) pg/mL; *p* < 0.001; *q* = 0.006), and IL-17A (3.7 (3.4, 4.0) pg/mL vs. 10.5 (6.0, 6.3) pg/mL; *p* = 0.002; *q* = 0.006). Additionally, cytokines of IL-15, TNF-α, and IL-1α showed comparable amounts between healthy implants and peri-implantitis (*p* < 0.05). Significant higher levels of chemokine CXCL2 (331.7 (214.1, 672.8) pg/mL vs. 1220.5 (726.3, 1880.0) pg/mL; *p* < 0.001; *q* = 0.002) were found in the PICF of peri-implantitis implants compared to healthy implants, with a fold change of 3.4. The cytokine of G-CSF also showed a significant difference between healthy implants sites and peri-implantitis sites (73.9 (32.8, 99.4) pg/mL vs. 350.7 (148.2, 477.9) pg/mL, *p* < 0.001; *q* = 0.001), with a fold change of 4.0. Additionally, chemokines levels of IL-8, RANTES, and MCP-1, and growth factor levels of VEGF, PDGF-AB/BB, and FGF-2 were highly expressed in the PICF of peri-implantitis compared to healthy implants (*p* < 0.05).

### 3.4. Correlations between Clinical Indexes and Microorganisms or Cytokines

**Figure 3:**
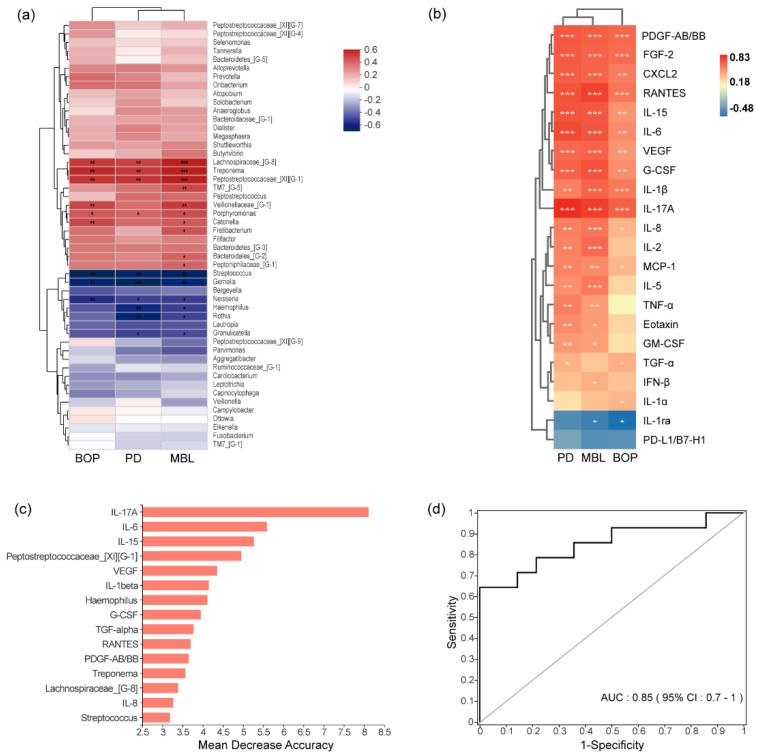
The heatmap shows the correlations between clinical indexes (PD, BOP, MBL) and the top 50 genera (**a**) and the differential cytokines between healthy implants and peri−implantitis (**b**) analyzed with Spearman’s correlation coefficient. The top 15 genera and cytokines of the optimal model were constructed by random forest classification (**c**). The receiver operating characteristic (ROC) curve was used to evaluate the constructed model (**d**). * *p* < 0.05, ** *p* < 0.01, *** *p* < 0.001. PD, probing depth; BOP, bleeding of probing; MBL, marginal bone loss; AUC, the area under the curve.

The genera of *Lachnospiraceae G-8*, *Treponema*, *Peptostreptococcus* *XIG-1*, and *Porphyromonas* had positive correlations with PD, BOP, and MBL (*r* > 0.4, *p* < 0.05), and the genera of *Streptococcus*, *Gemella*, and *Neisseria* had negative correlations with these clinical indexes (*r* < −0.4, *p* < 0.05, Figure 3a). A total of 10 out of 22 cytokines were positively correlated with PD, BOP, and MBL (*r* > 0.4, *p* < 0.05), including IL-1β, IL-6, IL-17A, IL-15, VEGF, PDGF-AB/BB, FGF-2, G-CSF, CXCL2, and RANTES. Cytokines of IL-17A, RANTES, and G-CSF were highly correlated with MBL (*r* = 0.77, *r* = 0.76, *r* = 0.72, *p* < 0.05). IL-17A was the cytokine with the highest correlation with those clinical indexes (PD, BOP, MBL) (PD, *r* = 0.83; BOP, *r* = 0.67; MBL, *r* = 0.77, *p* < 0.05). IL-1ra was negatively correlated with those clinical indexes (PD, *r* = −0.35; BOP, *r* = −0.48; MBL, *r* = −0.40, *p* < 0.05, Figure 3b).

### 3.5. Combined Diagnostic Ability of Microbiota and Cytokines

A random forest classification model was constructed for differentiating healthy implants and peri-implantitis relied on the relative abundance of bacterial populations and cytokines in the PICF. We determined the optimal model with the top 15 biomarkers, including the genera of *Peptostreptococcaceae XIG-1*, *Haemophilus*, *Treponema*, *Lachnospiraceae G-8*, and *Streptococcus*, as well as cytokines of IL-17A, IL-6, IL-15, VEGF, IL-1β, G-CSF, TGF-α, RANTES, PDGF-AB/BB, and IL-8. The ROC for the model had an area under the curve (AUC) of 0.85 (Figure 3c,d).

## 4. Discussions

Peri-implantitis is an inflammatory disease caused by microbial dysbiosis and an excessive host immune response. To the best of our knowledge, this is the first study to use a high-throughput approach to evaluate both microbiota and cytokine levels in the PICF from clinically healthy implants and peri-implantitis of the same individuals. In this study, microbiota composition and cytokines were significantly different between healthy implants and peri-implantitis, and the genera of *Peptostreptococcaceae XIG-1*, *Haemophilus*, and *Treponema*, as well as cytokines such as IL-17A, IL-6, IL-15, and IL-1β, might facilitate the diagnosis of peri-implantitis.

Healthy implants and peri-implantitis harbored different characteristics of the submucosal microbiome. In this study, microbial richness did not show a significant difference, while microbial diversity of peri-implantitis was higher compared to healthy implants. These results are consistent with previous studies [11]. Meanwhile, the composition of the submucosal microbiome associated with peri-implantitis has been a vital concern for understanding the pathology of this disease. Some common pathogens, including *Porphyromonas gingivalis*, *Tannerella forsythia*, *Prevotella intermedia*, and *Fusobacterium nucleatum*, have been detected in clinical samples of peri-implantitis reported by previous systematic reviews [24]. Nevertheless, a matched-pair study of healthy and diseased implants of the same individuals needed to be emphasized for the reason that it could better control biological variability and confounding factors [13], which was adopted by our study. This study showed that the genera of *Porphyromonas*, *Treponema*, *Filifactor*, and *Fretibacterium* accounted for the main part of the submucosal microbiome of peri-implantitis, while the genera of *Neisseria*, *Streptococcus*, *Haemophilus*, and *Rothia* occupied the microbial community in healthy implants. Only a few studies used 16S rRNA gene sequencing surveys of these two groups of the same individuals. Al-Ahmad et al. [10] showed similar results by collecting submucosal microbiome samples from ten individuals, indicating that some anaerobic Gram-negative pathogens seemed to play an important role in peri-implantitis. Another study characterized the intra-oral single-site submucosal microbiota of healthy and diseased implant sites from eighteen patients and showed that there was no difference in species (OTU) composition between the two groups [25]. Meanwhile, the phenotype structures of the microbiome showed that Gram-negative and anaerobic species were significantly enriched in peri-implantitis, while Gram-positive and aerobic species were abundant in the healthy implants. These results suggest that the alteration of microbiota composition in the submucosal sites might reflect the ecological shift, and the overgrowth of some potentially pathogenic bacteria might increase the host’s chances of developing peri-implantitis.

In addition to microorganisms as initial factors, host immunity is another crucial factor in the progression of peri-implantitis [26]. In this study, pro-inflammatory cytokines of IL-1β, IL-6, IL-17A, and TNF-α were significantly higher in peri-implantitis than those in healthy implants, and this result is consistent with another study [27]. Milinkovic et al. [28] also reported higher relative gene expression levels of IL-6, IL-17, IL-1β, and TNF-α in 50 peri-implantitis samples compared to 35 healthy implant samples, and significantly higher protein concentrations of IL-6 and IL-17 were detected in 27 peri-implantitis samples in comparison to 27 healthy implant samples. However, Hentenaar et al. [29] found that IL-1β was significantly elevated in peri-implantitis sites, while a significant difference in the levels of TNF-α and IL-6 was failed to be found between healthy and diseased implants from 20 healthy implants (N = 17 patients) and 20 implants with peri-implantitis (N = 19 patients). Persegani et al. [30] showed that IL-1β was significantly higher in shallow peri-implantitis sites compared to mucositis from 22 total edentulous. Severino et al. [31] demonstrated that higher expression of IL-17 was found in peri-implantitis compared to healthy implants; however, there was no significant difference when comparing the levels of IL-6 from 14 peri-implantitis samples and 11 healthy implant samples using the enzymatic immunosorbent assay (ELISA). Teixeira et al. [32] found that there was no significant difference in the expression of Th17-related cytokines between peri-implant mucositis and peri-implantitis. The cause of different results may be due to possible differences in disease definition, subject-to-subject variation, and different methods of sample collection or analysis. Otherwise, a systematic review concluded that the IL-1β, IL-6, IL-17, and TNF-α were the most frequently reported pro-inflammatory mediators associated with peri-implantitis [14]. Another two systemic reviews [16] presented that pro-inflammatory cytokines in PICF, such as IL-1β, TNF, and IL-6, were significantly elevated in peri-implantitis and could be used as adjunct tools to clinical parameters to differentiate healthy implants from peri-implantitis.

Chemokines are small heparin-binding proteins that direct the movement of circulating leukocytes to sites of inflammation or injury [26]. In this study, chemokines of CXCL2, IL-8, MCP-1, Eotaxin, and RANTES were significantly higher in peri-implantitis than those in healthy implant sites. CXCL2 plays an important role in the process of inflammatory response and damage repair, which is mainly generated by monocytes and macrophages, and could attract neutrophils to the inflammatory site [33]. An animal study showed a higher expression of CXCL2 in rat peri-implant soft tissue than in oral mucosa tissue during wound healing [34]. Our study was the first clinical investigation to find a higher level of CXCL2 in PICF of peri-implantitis. Further study is required to investigate the function of CXCL2 in peri-implantitis.

In addition, the cytokine levels of VEGF, PDGF-AA/BB, FGF-2, G-CSF, and GM-CSF in peri-implantitis sites were significantly higher than those in healthy implant sites. VEGF has the function of increasing angiogenesis and vascular permeability [35]. A histological study showed that VEGF was highly expressed in peri-implantitis soft tissue, and it was also positively correlated with peri-implant pocket depth [36]. G-CSF is a key regulator of neutrophil production [37]. A study reported that anti-G-CSF antibody administration could mitigate alveolar bone resorption in the experimental periodontitis model, inferring that G-CSF might be one of the essential immune factors that mediate bone loss in periodontitis [38]. However, a recent cross-sectional study failed to find a significant difference of G-CSF in the PICF between healthy and diseased implants [29], probably because of the influence of individual differences. Moreover, our results show that G-CSF had a highly significant correlation with MBL. These results indicate that G-CSF might induce an excessive inflammatory response and eventually cause alveolar bone loss, and its mechanism is worth further exploration.

Clinical conditions, including inflammatory immune response and bone loss, were strongly associated with the peri-implant microbiota. In this study, the genera of *Lachnospiraceae G-8*, *Treponema*, and *Peptostreptococcaceae XIG-1* had a positive correlation with these three clinical indexes: BOP, PD, and MBL. Recently, Barbagallo et al. [39] pointed out that *Peptostreptococcaceae XIG-1* and *Peptostreptococcaceae XIG-6* were over-abundant in peri-implantitis compared to healthy teeth. Meanwhile, Wang et al. [40] found that the genus of *Peptostreptococcaceae XIG-9* was significantly more abundant at peri-implant mucositis sites with suppuration. In our study, *Peptostreptococcaceae XIG-1* was detected with significantly higher relative abundance at peri-implantitis sites, which indicates that species of *Peptostreptococcus* reside in the healthy implant site as commensals, but may be opportunistic pathogens with potential correlations with peri-implantitis. The genus of *Treponema* was also more abundant in biofilms from peri-implantitis compared to those from healthy implants. Research showed that *Treponema* was at the forefront of established infections, and their presence exacerbates the damage to the supporting tissues [41]. These microbiota may play a vital role in the occurrence and development of peri-implantitis diseases, and have strong correlation with the clinical index. Shi et al. [42] compared the microbiome of peri-implant mucositis and peri-implantitis, concluding that an increase in MBL was associated with submucosal microbial dysbiosis. A previous study by Kröger and colleagues also showed that the PD had a substantial relevance to the microbiome of the peri-implantitis sites, highlighting an increased dysbiosis in deeper pockets [43]. Additionally, these clinical indexes were also associated with cytokine levels in peri-implantitis in this study, which showed that IL-1β, IL-6, IL-17A, VEGF, CXCL2, and G-CSF had a positive correlation with these three clinical indexes. A narrative review provided evidence for correlations between MBL and cytokines, including IL-1β, IL-6, IL-17, TNF-α, and VEGF, indicating cytokines might enhance osteoclast formation and bone resorption [44]. Altogether, the results of correlations between microbiome and cytokines and the clinical indexes revealed the dysbiosis of the microbiome along with the occurrence of peri-implantitis, followed by the significant change of associated cytokines, which provides a theoretical basis for future mechanism research.

Given the above clinical relevance, it is very important to establish a combined model of microorganisms and factors to distinguish peri-implantitis from healthy implants, which provides more basis for clinical diagnosis and targeted treatment. Wang et al. [45] explored the profiles of the microbiome and PICF biomarkers with qPCR analysis from healthy and peri-implantitis sites, and the results showed that *T. denticola* combined with IL-1β, VEGF, and TIMP-2 PICF levels could diagnose diseased sites. Since then, there were no data about the combination of microbiome and cytokines to differentiate diseased and healthy conditions. This study utilized the random forest algorithm, which is a decision tree-based machine learning algorithm for classification that accounts for non-linear data and interactions among features and includes internal cross-validation to prevent overfitting [46]. With this method, we found that the genus of *Peptostreptococcaceae XIG-1*, *Treponema*, *Lachnospiraceae G-8*, *Haemophilus*, and *Streptococcus* combined with some pro-inflammation cytokines, including IL-6, IL-17A, and IL-1β, and growth factors, including VEGF and G-CSF, and chemokines including RANTES, could distinguish the disease status. However, the classification model needs validation in a large population, and further study could explore microbiological and immune-associated treatment to impede the progress of peri-implantitis.

The advantage of this study is that it evaluates the microorganism and cytokines levels simultaneously, which provides thorough profiling of the peri-implant microenvironment. Moreover, in this study, a total of 170 patients were screened and 14 patients with at least one healthy implant and one peri-implantitis implant were ultimately recruited, and a within-one-subject design could better control individual differences and perhaps eliminate the effect of confounding factors from different persons. However, that also results in a small sample size, which is the main limitation. Moreover, some risk factors need to be taken into consideration: many studies reported poor oral hygiene, smoking, less keratinized tissue, and emergence angle of more than 30 degrees combined with a convex emergence profile of the abutment/prosthesis could increase the risk for peri-implantitis [47,48,49]. In our study, statistical analysis was conducted to compare factors, including implant position, implant diameter, implant length, bone augmentation, and prosthetic type, and no significant difference was found between healthy implants and peri-implantitis. However, the small sample size had limitations, and prospective, multicenter cohort studies are needed to further investigate risk factors in peri-implantitis. Meanwhile, the oral cavity is a dynamic and complicated environment that harbors more than 700 bacteria species [50]. Bacteria and cytokines in healthy implants might be different due to the different periodontal status of the full mouth and adjacent teeth or implant, which is an interesting topic that needs further investigation. The following constraints should be also noted. More samples need to be included because the cross-sectional study does not allow any claim of causality. Prospective studies with a larger cohort will provide insights into the utility of diagnosing and monitoring peri-implant diseases.

## 5. Conclusions

In the same individual, peri-implantitis sites harbored more abundance of *Treponema*, *Peptostreptococcaceae XIG-1*, *Porphyromonas*, and *Lachnospiraceae G-8*, as well as cytokines of IL-6, IL-17A, G-CSF, RANTES, and IL-1β, than healthy implants sites. Furthermore, a classification model picked up biomarkers such as *Peptostreptococcaceae XIG-1*, *Treponema*, IL-6, and IL-17A to distinguish healthy implants from peri-implantitis. A comprehensive profile of the peri-implant microenvironment without individual differences may better assist in exploring the pathophysiological underpinnings, diagnosis, and therapeutic strategies for peri-implantitis.

## Figures and Tables

**Table 1:** Clinical variables of this study at implant level (N = 14).

	Peri-Implantitis	Healthy	*p*-Values *
mPD (mm, mean ± SD)	5.2 ± 1.3	2.5 ± 0.5	**0.001**
mBOP (%, mean ± SD)	65.5 ± 28.1	0	**0.001**
mMBL (mm, mean ± SD)	3.4 ± 1.4	0	**0.001**
PICF volume (μL, mean ± SD)	0.3 ± 0.04	0.2 ± 0.04	**0.001**
KGW (<2 mm/≥2 mm)	4/10	4/10	1.0
Implant position (Maxillary/Mandibular) (Anterior/Posterior)	9/5 5/9	8/6 1/13	0.7 0.2
Diameter (mm, mean ± SD)	4.0 ± 0.6	4.1 ± 0.5	0.6
Length (mm, mean ± SD)	11.7 ± 1.2	11.3 ± 1.1	0.3
Bone augmentation (GBR/sinus lift/none)	1/5/8	3/3/8	0.5
Prosthetic types (single crown/bridge)	6/8	9/5	0.3

*Note*: Wilcoxon signed-rank test is used for quantitative data, and a chi-square test is used for enumeration data. * Significant differences with *p*-values < 0.002 are marked with boldface. Abbreviations: mPD, mean pocket depth; mBOP, mean bleeding on probing; mMBL, mean marginal bone loss; PICF, peri-implant crevicular fluid; KGW, keratinized gingiva width; GBR, guided bone regeneration.

**Table 2:** Cytokine levels in PICF between healthy implants and peri-implantitis.

	Concentration [pg/mL, Median (Q1, Q3)]		Quantity [pg, Median (Q1, Q3)]				
Cytokines	Healthy	Peri-Implantitis	Healthy	Peri-Implantitis	*p*-Value	*q*-Value	log_2_ (fc)
G-CSF	73.9 (32.8, 99.4)	350.7 (148.2, 477.9)	4.4 (2.0, 6.0)	21.0 (8.9, 28.7)	**<0.001**	**0.001**	**2.01**
IL-15	1.3 (1.1, 1.5)	2 (1.6, 2.2)	0.1 (0.1, 0.1)	0.1 (0.1, 0.1)	**<0.001**	**0.001**	0.65
PDGF-AB/BB	1.8 (1.6, 2.1)	3.4 (2.2, 3.6)	0.1 (0.1, 0.1)	0.2 (0.1, 0.2)	**<0.001**	**0.001**	**1.12**
IL-8	2113 (864.3, 2749.0)	3533 (2363.5, 3697.8)	126.8 (51.9, 164.9)	212.0 (141.8, 221.9)	**<0.001**	**0.001**	0.76
CXCL2	331.7 (214.1, 672.8)	1220.5 (726.3, 1880.0)	19.9 (12.8, 40.4)	73.2 (43.6, 112.8)	**<0.001**	**0.002**	**1.77**
VEGF	324.7 (177.9, 409.2)	945.7 (456.8, 1343.0)	19.5 (10.7, 24.6)	56.7 (27.4, 80.6)	**<0.001**	**0.002**	**1.61**
IL-2	14.9 (9.4, 17.9)	19.8 (17.3, 21.0)	0.9 (0.6, 1.1)	1.2 (1.0, 1.3)	**<0.001**	**0.002**	0.47
FGF-2	15.5 (7.3, 19.1)	37.3 (23.4, 61.7)	0.9 (0.4, 1.1)	2.2 (1.4, 3.7)	**<0.001**	**0.003**	**2.50**
IL-1β	1403.0 (735.8, 2083.8)	4311 (2388.5, 5502.5)	84.2 (44.2, 125.0)	258.7 (143.3, 330.2)	**<0.001**	**0.004**	**1.41**
IL-17A	3.7 (3.0, 4.4)	10.5 (6.6, 25)	0.2 (0.2, 0.3)	0.6 (0.4, 1.5)	0.002	**0.006**	**2.41**
RANTES	108.7 (99.6, 119.4)	162.4 (122.2, 191.7)	6.5 (6.0, 7.1)	9.7 (7.3, 11.5)	0.002	**0.006**	0.65
IL-6	5.4 (4.5, 8.2)	38.3 (16.2, 221.7)	0.3 (0.3, 0.5)	2.3 (1.0, 13.3)	0.002	**0.006**	**6.82**
IL-5	1.9 (1.8, 2.1)	2.4 (2.1, 2.5)	0.1 (0.1, 0.1)	0.1 (0.1, 0.2)	0.004	0.014	0.46
TGF-α	52.7 (37.3, 75.4)	72.8 (65.7, 88.1)	3.2 (2.2, 4.5)	4.3 (4.0, 5.3)	0.006	0.018	0.64
Eotaxin	16.3 (13.7, 18.1)	19.5 (16.5, 22.8)	1.0 (0.8, 1.1)	1.2 (1.0, 1.4)	0.006	0.018	0.43
TNF-α	8.6 (4.7, 9.1)	11.3 (6.5, 40.4)	0.5 (0.3, 0.5)	0.7 (0.4, 2.4)	0.007	0.018	**1.75**
IL-1α	2593.5 (1814.7, 4590.0)	6263.0 (4030.0, 8863.0)	155.6 (108.9, 275.4)	375.8 (241.8, 531.8)	0.011	0.028	0.80
IFN-β	1.3 (1.1, 1.9)	2.4 (1.6, 3.4)	0.1 (0.1, 0.1)	0.1 (0.1, 0.2)	0.014	0.033	0.87
IL-1ra	18,813.0 (16,475.0, 20,163.0)	15,480.0 (14,761.0, 17,251.0)	1128.8 (988.5, 1209.8)	928.8 (885.7, 1035.1)	0.013	0.033	−0.10
MCP-1	50.0 (29.7, 115.4)	176.0 (72.5, 415.1)	3.0 (1.8, 7.0)	10.6 (4.3, 24.9)	0.017	0.037	**1.47**
GM-CSF	10.2 (8.0, 18.9)	25.7 (10.3, 83.4)	0.6 (0.5, 1.1)	1.5 (0.6, 5.0)	0.020	0.041	**2.09**
PD-L1/B7-H1	219.0 (133.0, 248.8)	141.1 (96.5, 215.3)	13.1 (8.0, 15.0)	8.5 (5.8, 12.9)	0.020	0.041	−0.44
IL-12p70	6.8 (6.4, 7.1)	7.23 (6.6, 8.3)	0.4 (0.4, 0.4)	0.4 (0.4, 0.5)	0.041	0.077	0.35
IL-3	13.8 (6.2, 27.4)	5.7 (4.7, 9.6)	0.8 (0.4, 1.6)	0.3 (0.3, 0.6)	0.042	0.077	**−1.01**
IL-17E	9.8 (9.5, 10.6)	11.8 (10.8, 13.7)	0.6 (0.6, 0.6)	0.7 (0.6, 0.8)	0.048	0.081	0.28
TRAIL	51.7 (34.7, 71.4)	71.1 (45.7, 120.6)	3.1 (2.1, 4.3)	4.3 (2.7, 7.2)	0.049	0.081	0.62
IL-7	1.1 (1.0, 1.2)	1.3 (1.2, 1.0)	0.1 (0.1, 0.1)	0.1 (0.1, 0.1)	0.046	0.081	0.31

*Note*: Wilcoxon signed-rank paired test is used for the analysis of levels in PICF between the healthy implants and peri-implantitis. Significant differences with *p* < 0.001 are marked with boldface. Significant differences with *q* < 0.01 are marked with boldface. Significant fold changes (log2(fc) > 1) are marked with boldface. Abbreviations: PICF, peri-implant crevicular fluid; Q1, quarter; Q3, three quarters.

## Data Availability

Not applicable.

## Supplementary Materials

The following supporting information can be downloaded at: https://www.mdpi.com/article/10.3390/jcm11195817/s1.
