# Supplementary material for: The Characteristics of Microbiome and Cytokines in Healthy Implants and Peri-Implantitis of the Same Individuals
Source: J Clin Med. 2022 Sep 30;11(19):5817. doi: 10.3390/jcm11195817 (PMC9572122; doi:10.3390/jcm11195817)
Supplement: Supplementary file 1 [file jcm-11-05817-s001.zip › jcm-1875462-supplementary/Supplementary files/Table S1.pdf]

Table S1. Demographic details and general information of the 14 patients

Patient	Gender	Age	Number of implants	Number of remaining teeth	Full-mouth mPLI	Full-mouth mGI	Full-mouth mPD	Full-mouth mBOP(%)	Smoker	Periodontitis	Periodontitis stage	Periodontitis grade	Periodontitis Status(controlled)
1	Male	65	7	14	2	1	2.90	21.00	Yes	Yes	IV	C	Yes
2	Female	67	2	26	1	1	2.63	18.00	NO	Yes	I	A	Yes
3	Male	62	8	18	1	1	2.66	25.00	Yes	Yes	IV	C	Yes
4	Male	53	2	26	1	1	2.77	13.10	NO	Yes	I	A	Yes
5	Male	66	7	20	2	2	2.28	25.00	NO	Yes	I	A	Yes
6	Female	33	2	26	2	2	2.96	55.95	NO	Yes	I	A	No
7	Male	59	8	7	1	1	3.5	10.00	Yes	Yes	II	A	Yes
8	Male	46	4	23	1	1	2.49	23.72	Yes	NO			Yes
9	Male	54	11	11	1	2	3.23	15.08	Yes	Yes	IV	C	Yes
10	Female	64	11	10	2	1	2.91	15.56	NO	Yes	II	A	Yes
11	Female	47	8	16	1	1	2.3	31.94	NO	NO			Yes
12	Male	48	6	17	2	2	4.07	57.97	NO	Yes	III	B	No
13	Female	57	3	24	1	1	2.52	1.28	NO	Yes	I	A	Yes
14	Female	65	7	17	1	1	2.5	2.27	NO	Yes	IV	B	Yes

Abbreviation: mPLI, mean plaque index; mGI, mean gingival index; mPD, mean pocket depth; mBOP, mean bleeding on probing
