# Supplementary material for: The Characteristics of Microbiome and Cytokines in Healthy Implants and Peri-Implantitis of the Same Individuals
Source: J Clin Med. 2022 Sep 30;11(19):5817. doi: 10.3390/jcm11195817 (PMC9572122; doi:10.3390/jcm11195817)
Supplement: Supplementary file 1 [file jcm-11-05817-s001.zip › jcm-1875462-supplementary/Supplementary files/Table S2.pdf]

Table S2. Demographic details and general information of the 28 implants

Patient	Implant status	Implant position	Time in function(months)	mPD (mm)	mBOP(%)	mMBL (mm)	KGW (mm)	Implant brand	Diameter (mm)	Length (mm)	Bone augmentation	Type of restoration
1	Healthy	45	41.4	2.16	0	0	1	Straumann	4.1	10	No	Singles crown
1	Peri-implantitis	21	28.6	4.67	50	2.65	6	straumann	3.3	14	No	Bridge
2	Healthy	24	101.1	3.00	0	0	3	Straumann	3.1	10	No	Singles crown
2	Peri-implantitis	27	98.9	6.00	100	3.21	3	Straumann	4.8	10	Sinus lift	Singles crown
3	Healthy	44	64.7	2.50	0	0	3	Straumann	4.8	10	No	Bridge
3	Peri-implantitis	32	64.7	4.67	100	2.58	5	Straumann	3.3	14	No	Bridge
4	Healthy	26	90.2	2.83	0	0	5	Ankylos	4.5	11	No	Singles crown
4	Peri-implantitis	44	73.6	4.33	50	2.78	1	Straumann	4.8	8	No	Bridge
5	Healthy	26	28.3	2.67	0	0	2	Straumann	4.8	10	Sinus lift	Bridge
5	Peri-implantitis	36	28.3	3.50	50	1.05	3	Straumann	4.1	12	No	Bridge
6	Healthy	46	60.8	2.00	0	0	2	Ankylos	3.5	11	No	Singles crown
6	Peri-implantitis	36	60.8	5.50	100	2.32	2	Ankylos	3.5	11	No	Singles crown
7	Healthy	35	97.6	3.00	0	0	0	Straumann	4.1	12	GBR	Bridge
7	Peri-implantitis	26	94.9	6.50	100	4.75	2	Straumann	4.8	12	Sinus lift	Bridge
8	Healthy	25	49.2	2.67	0	0	5	Nobel	4.3	12	No	Singles crown
8	Peri-implantitis	16	64.7	4.00	50	5.56	4	Nobel	4.3	12	No	Singles crown
9	Healthy	24	72.5	2.67	0	0	3	Straumann	4.1	12	GBR	Singles crown
9	Peri-implantitis	13	72.5	3.67	33.3	3.59	5	Straumann	4.8	12	No	Bridge
10	Healthy	21	76.5	2.83	0	0	3	Straumann	3.3	14	GBR	Bridge
10	Peri-implantitis	25	76.5	6.00	83.3	6.10	0	Straumann	4.1	12	Sinus lift	Bridge
11	Healthy	37	85.8	2.33	0	0	1	Ankylos	4.5	11	No	Bridge
11	Peri-implantitis	35	85.8	3.67	83.3	2.25	0	Ankylos	3.5	11	No	Bridge
12	Healthy	44	78.9	2.83	0	0	5	Ankylos	3.5	11	No	Singles crown
12	Peri-implantitis	32	78.9	8.33	66.7	2.71	2	Ankylos	3.5	11	No	Singles crown
13	Healthy	25	81.2	2.17	0	0	2	Straumann	4.1	12	Sinus lift	Singles crown
13	Peri-implantitis	27	81.2	5.33	33.3	3.73	1	Straumann	4.1	12	Sinus lift	Singles crown
14	Healthy	26	83.8	1.33	0	0	1	Straumann	4.1	12	Sinus lift	Singles crown
14	Peri-implantitis	17	87.7	6.17	33.3	3.76	0	Straumann	4.8	10	Sinus lift	Bridge

Abbreviation: mPD, mean pocket depth; mBOP, mean bleeding on probing; mMBL, mean marginal bone loss; KGW, keratinized gingiva width
