# Supplementary material for: The Characteristics of Microbiome and Cytokines in Healthy Implants and Peri-Implantitis of the Same Individuals
Source: J Clin Med. 2022 Sep 30;11(19):5817. doi: 10.3390/jcm11195817 (PMC9572122; doi:10.3390/jcm11195817)
Supplement: Supplementary file 1 [file jcm-11-05817-s001.zip › jcm-1875462-supplementary/Supplementary files/Table S3.pdf]

Table S3. Cytokine levels in PICF between healthy implants and peri-implantitis.

	**Concentration** [pg/ml, median (Q1, Q3)]		**Quantity** [pg, median (Q1, Q3)]				
**Cytokines**	**Healthy**	**Peri-implantitis**	**Healthy**	**Peri-implantitis**	***p* value**	***q* value**	**log_2 (fc)**
Granzyme B	171.8 (80.2, 194.9)	200.4 (111.7, 274.7)	10.3 (4.8, 11.7)	12.0 (6.7, 16.5)	0.07	0.11	0.79
GRO alpha	1450.1 (1106.5, 3209.5)	2896.0 (1798.8, 3347.2)	87.0 (66.4, 192.6)	173.8 (107.9, 200.8)	0.07	0.11	0.33
IL-13	17.8 (17.4, 19.2)	19.2 (17.7, 20.7)	1.1 (1.0, 1.2)	1.2 (1.1, 1.2)	0.11	0.17	0.19
PDGF-AA	27.9 (15.0, 42.3)	38.5 (28.5, 63.0)	1.7 (0.9, 2.5)	2.3 (1.7, 3.8)	0.15	0.22	0.71
MIP-1alpha	43.4 (30.5, 52.3)	46.3 (31.9, 68.7)	2.6 (1.8, 3.1)	2.8 (1.9, 4.1)	0.27	0.36	0.97
IFN-gamma	9.8 (5.5, 10.5)	6.1 (3.9, 9.9)	0.6 (0.3, 0.6)	0.4 (0.2, 0.6)	0.27	0.36	-0.17
MIP-1beta	336.4 (118.8, 437.9)	327.5 (239.7, 580.9)	20.2 (7.1, 26.3)	19.6 (14.4, 34.8)	0.29	0.37	1.14
IP-10	14.1 (6.0, 19.7)	9.7 (2.8, 25.2)	0.8 (0.4, 1.2)	0.6 (0.2, 1.5)	0.29	0.37	-1.01
IL-10	23.6 (20.5, 29.1)	20.5 (18.4, 31.7)	1.4 (1.2, 1.7)	1.2 (1.1, 1.9)	0.53	0.64	0.18
IL-33	10.6 (9.0, 12.2)	12.1 (9.4, 13.7)	0.6 (0.5, 0.7)	0.7 (0.6, 0.8)	0.54	0.64	0.17
IL-4	0.2 (0.2, 0.3)	0.3 (0.3, 0.3)	0.01 (0.01, 0.02)	0.02 (0.01, 0.02)	0.56	0.64	0.12
MIP-3alpha	13.1 (9.5, 18.9)	15.2 (9.5, 18.2)	0.8 (0.6, 1.1)	0.9 (0.6, 1.1)	0.62	0.69	0.42
Flt-3 Ligand	29.0 (24.4, 43.8)	30.8 (24.2, 44.6)	1.7 (1.5, 2.6)	1.8 (1.5, 2.7)	0.63	0.69	0.34
IFN-alpha	2.2 (1.7, 3.0)	1.9 (1.6, 4.0)	0.1 (0.1, 0.2)	0.1 (0.1, 0.2)	0.68	0.72	0.61

MIP-3beta	2.7 (2.0, 3.6)	2.1 (1.8, 3.8)	0.2 (0.1, 0.2)	0.1 (0.1, 0.2)	0.86	0.87	0.04
CD40 Ligand	868.7 (497.4, 1146.2)	689.5 (471.4, 1015.0)	52.1 (29.8, 68.8)	41.4 (28.3, 60.9)	0.86	0.87	0
EGF	47.1 (32.5, 165.1)	38.0 (25.5, 100.4)	2.8 (2.0, 9.9)	2.3 (1.5, 6.0)	0.94	0.94	-0.5

Note: Wilcoxon signed-rank paired test is used for the analysis of levels in PICF between the healthy implants and peri-implantitis.
Abbreviation: PICF, peri-implant crevicular fluid; Q1, quarter; Q3, three quarters.
