# Supplementary figures and images for: The Characteristics of Microbiome and Cytokines in Healthy Implants and Peri-Implantitis of the Same Individuals
Source: J Clin Med. 2022 Sep 30;11(19):5817. doi: 10.3390/jcm11195817 (PMC9572122; doi:10.3390/jcm11195817)

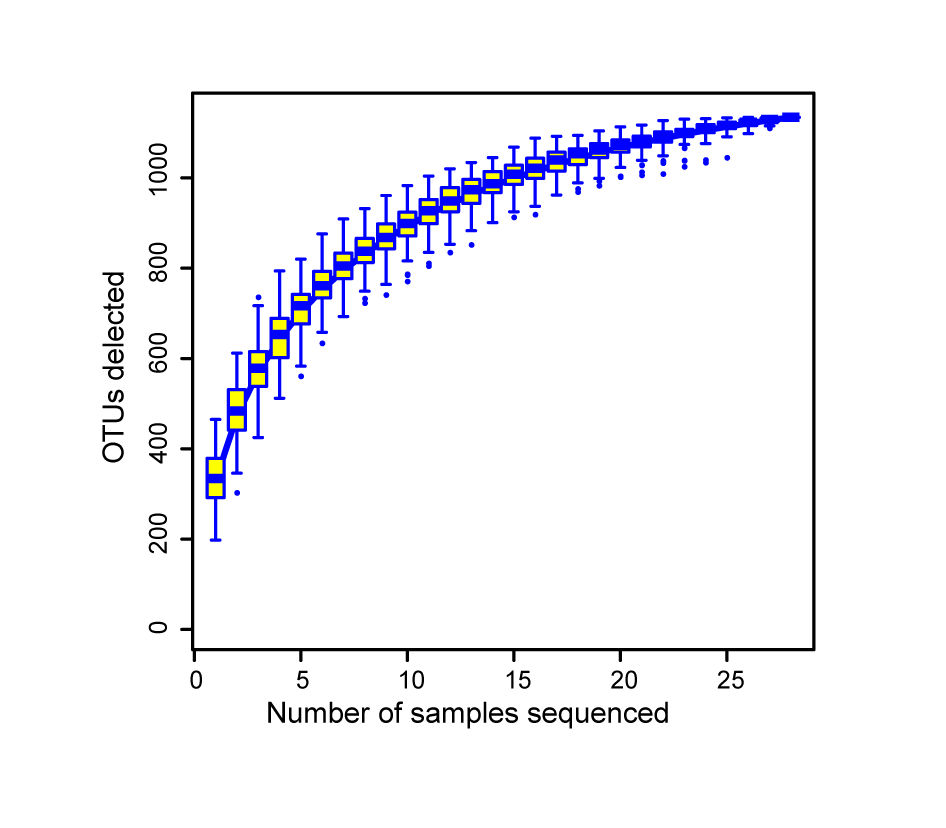

Supplement: Supplementary file 1 [file jcm-11-05817-s001.zip › jcm-1875462-supplementary/Supplementary files/Figure S1.tif]

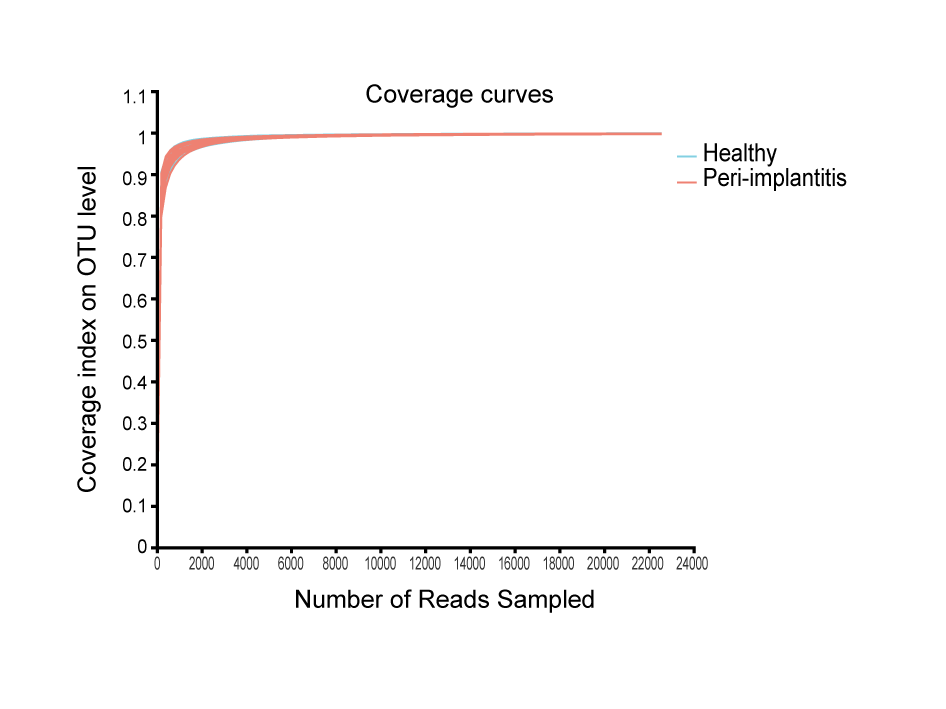

Supplement: Supplementary file 1 [file jcm-11-05817-s001.zip › jcm-1875462-supplementary/Supplementary files/Figure S2.tif]

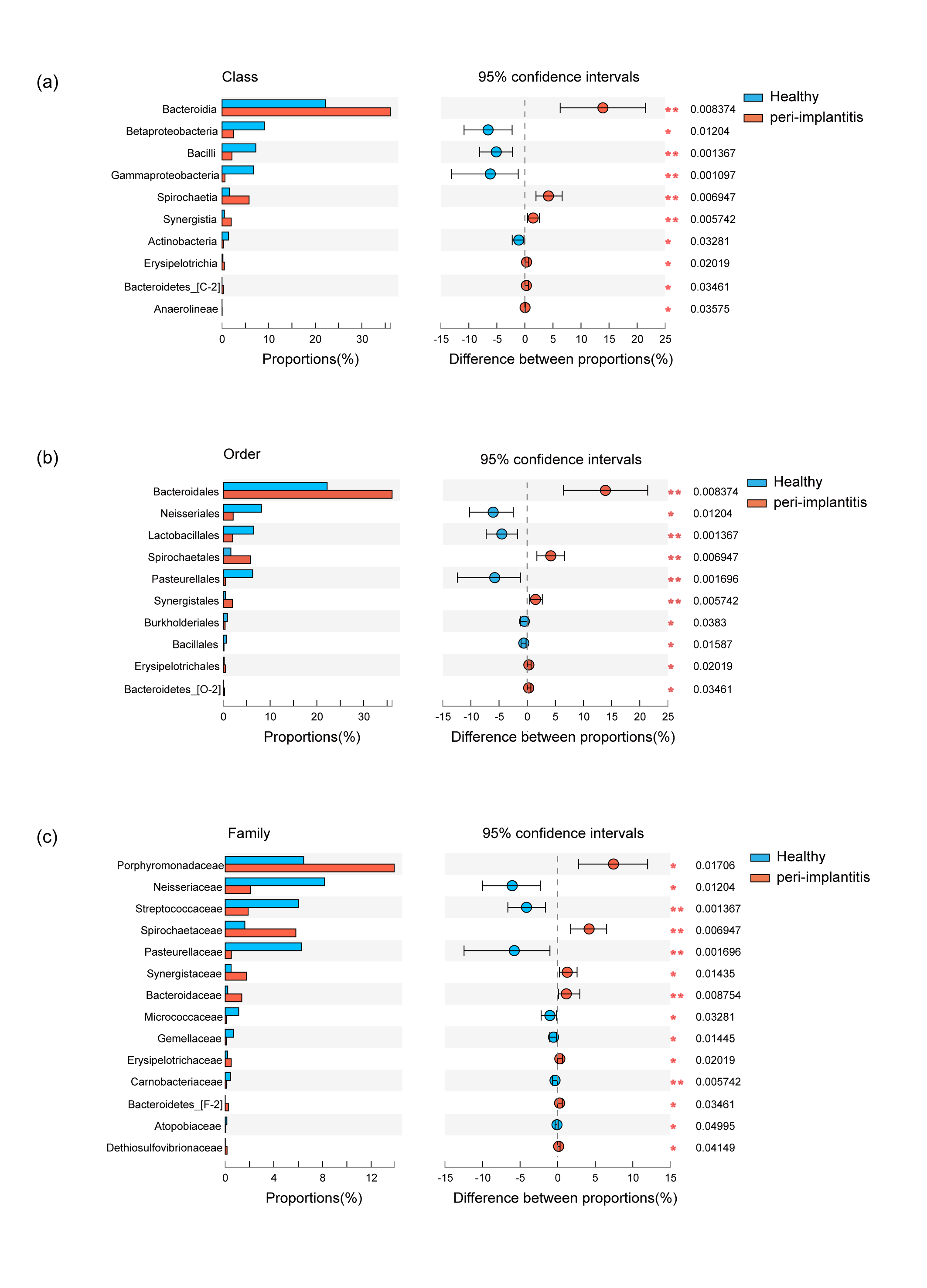

Supplement: Supplementary file 1 [file jcm-11-05817-s001.zip › jcm-1875462-supplementary/Supplementary files/Figure S3.tif]

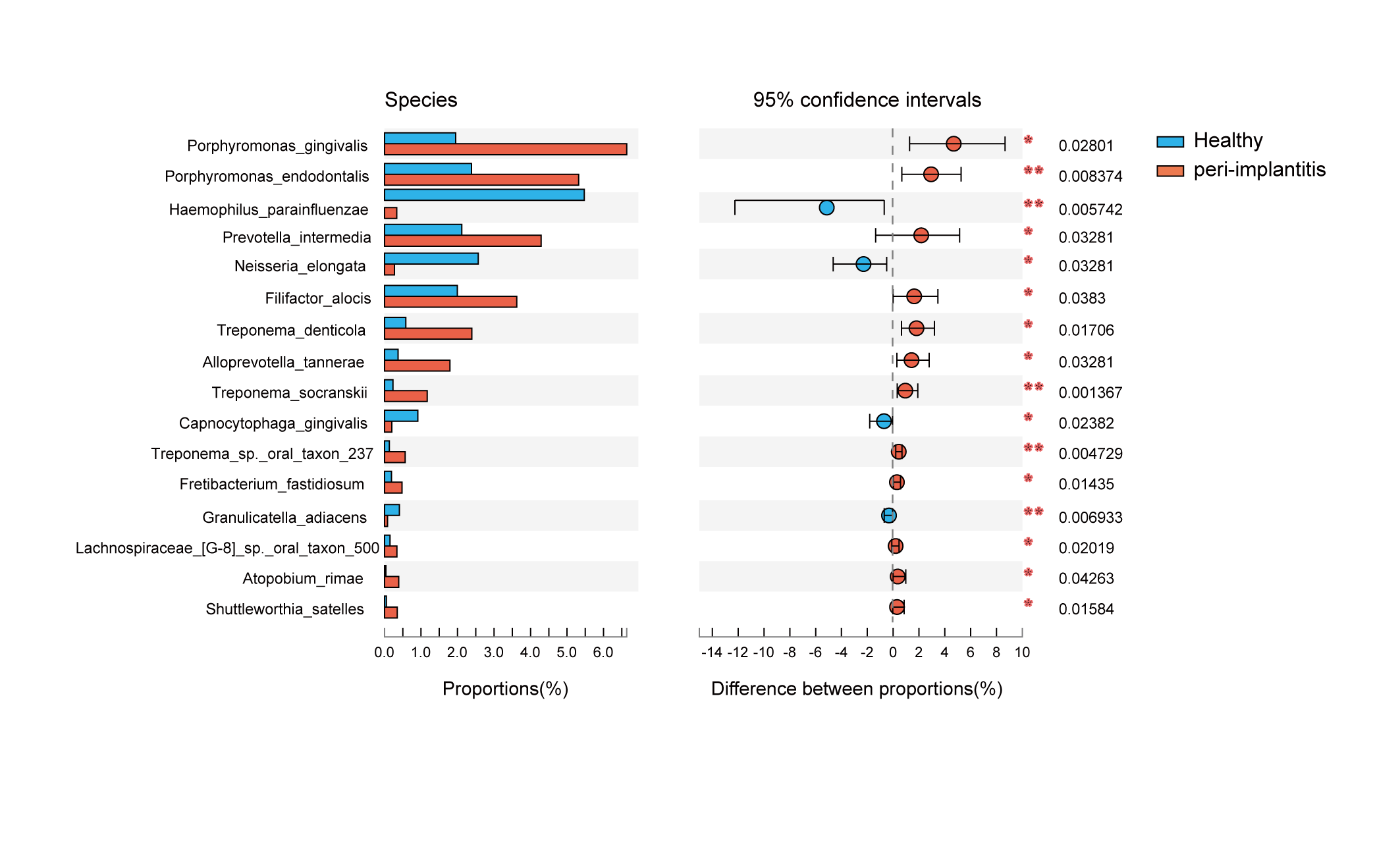

Supplement: Supplementary file 1 [file jcm-11-05817-s001.zip › jcm-1875462-supplementary/Supplementary files/Figure S4.tif]
